# Essential Oils for Complementary Treatment of Surgical Patients: State of the Art

**DOI:** 10.1155/2014/726341

**Published:** 2014-02-24

**Authors:** Susanna Stea, Alina Beraudi, Dalila De Pasquale

**Affiliations:** ^1^Medical Technology Laboratory, Istituto Ortopedico Rizzoli, Via di Barbiano 1/10, 40136 Bologna, Italy; ^2^Prometeo Laboratory, Istituto Ortopedico Rizzoli, Via di Barbiano 1/10, 40136 Bologna, Italy

## Abstract

Aromatherapy is the controlled use of plant essences for therapeutic purposes. Its applications are numerous (i.e., wellbeing, labour, infections, dementia, and anxiety treatment) but often they have not been scientifically validated. The aim of the present study is to review the available literature to determine if there is evidence for effectiveness of aromatherapy in surgical patients to treat anxiety and insomnia, to control pain and nausea, and to dress wound. Efficacy studies of lavender or orange and peppermint essential oils, to treat anxiety and nausea, respectively, have shown positive results. For other aspects, such as pain control, essential oils therapy has shown uncertain results. Finally, there are encouraging data for the treatment of infections, especially for tea tree oil, although current results are still inconclusive. It should also be considered that although they are, allergic reactions and toxicity can occur after oral ingestion. Therefore, while rigorous studies are being carried out, it is important that the therapeutic use of essential oils be performed in compliance with clinical safety standards.

## 1. Introduction

Patients undergoing surgery can benefit from complementary medicine treatments, such as acupuncture, relaxation techniques, massage, and soft manipulation, without putting a burden on the therapeutic plan but, on the contrary, relieving it.

Amongst complementary medicine treatments a particular attention is to be given to essential oils (EOs) treatments that, for their pleasantness and inexpensiveness, can result to be quite useful. Aromatherapy is often associated with other treatments, such as massage; therefore it is difficult to isolate their effect when applied topically. Nevertheless, there is some clinical scientific evidence in favour of EO use in various phases of pre- and postoperative treatment. It should be remarked that this approach is successful also in economically disadvantaged countries where the medium/low cost of this therapy can be supported by the national health system [1].

The mechanism of action of inhaled aromatherapy starts with the absorption of volatile molecules through the nasal mucosa. Odor molecules are then transformed into chemical signals, which move towards the olfactory bulb, and possibly other parts of the limbic system, interacting with the neuropsychological framework to produce characteristic physiological and psychological effects.

The aim of the present work is to illustrate the applications of aromatherapy to surgical patients, on the basis of scientific evidence.

## 2. Preoperative Anxiety

Preoperative anxiety is a common problem that patients undergoing surgery are facing. Surgical procedures, regardless of the difficulty of the intervention, can cause considerable apprehension, mainly reported as the fear of being unconscious, the operation itself, and pain when recovering from anesthesia.

Many anxious patients receive medications such as sedatives that may be associated with adverse side effects and reduce their capacity to actively and positively participate in post-op care. The most appropriate oils to treat anxiety are lavender and orange and there is a variety of literature on them.

Evidence of efficacy of EOs in randomized clinical trials (RCTs) is not definitive. Two meta-analyses performed in 2011 [2] and in 2012 [3] on RCTs, both analyzing papers published up to 2010, came to slightly different conclusions: the first analysis concluded that there were insufficient clinical trials examining the effects of aromatherapy in general among people with anxiety disorders as primary condition, but that aromatherapy can be considered as a safe and pleasant intervention in case of secondary anxiety.

The second analysis verified more specifically the effect of lavender EO and concluded that evidence for oral administration of lavender is promising but remains inconclusive.

One possible reason for doubtful results may be linked to essential oil source; there are indeed various types of lavender species used as an EO in clinical aromatherapy practice. Lavender oils include true lavender (*Lavandula angustifolia*), *lavender stoechas* (*Lavandula stoechas*), spike lavender (*Lavandula latifolia*), and lavandin (*Lavandula*  ×  *intermedia*) and this should be considered when efficacy is evaluated, as their chemical composition may differ considerably.

One of the major components of lavender EO is linalool, which has been demonstrated to act postsynaptically, possibly via the modulation of the activity of cyclic adenosine monophosphate (cAMP) [4]. In animal models, linalool has been found to inhibit GABA(A) binding receptor in the central nervous system inducing a relaxed state [5, 6]. Until recently, this activity had not been proven in human studies.

More recently, Schuwald gave further evidence of lavender EO mechanism of action using experimental models of low oral doses corresponding to dosages given in humans (80 mg/d) and demonstrating an inhibition of voltage-dependent calcium channels VOCCs [7].

Clinical evidence of the relaxing efficacy of lavender EO was obtained by Braden et al. [8] who enrolled 150 adult patients undergoing different types of surgery and then randomly assigned to either control (standard care), experimental (standard care plus EO lavandin, *Lavandula hybrida*), or sham (standard care plus jojoba oil) groups. Oils were sniffed and applied on the skin before surgery. Visual analog scales were used to assess anxiety on admission to preoperative suite and operating room transfer. It resulted in that the lavandin group showed significantly lower anxiety during operating room transfer.

The evidence of the efficacy of lavender essential oil was also confirmed by Kim et al. [9] who assessed stress and pain level of needle insertion in the preoperative area after inhalation of *Lavandula augustifolia* from a swab put in the oxygen mask.

Similar results were obtained by Lehrner et al. [10], in the waiting time for dental procedures; even if this cannot be considered as properly surgery, it represents an extremely “strong” emotional situation. 200 patients were either stimulated with ambient odor of orange (*Citrus sinensis*) or ambient odor of lavender. These conditions were compared to a music condition and a control condition (no odor and no music). It resulted in that both ambient odors of orange and lavender reduced anxiety and improved mood in patients waiting for dental treatment.

Orange essential oil diffused in the ambient demonstrated the capacity to reduce stress, measured as salivary cortisol and cardiac pulse, also in a different group of pediatric patients, during dental treatment [11].

The efficacy of *Citrus sinensis* has been proved in stressful conditions different from surgery but in very controlled and rigorous conditions (such as comparison to other smells or absence of other olfactive stimuli) also by Goes et al. who showed an acute anxiolytic activity of sweet orange aroma [12].

Evidence of the efficacy of other essential oils in anxiety control is modest with the exception of an interesting finding, by Hongratanaworakit, who observed a relaxing effect of *Rosa damascena* EO administered to volunteers by transcutaneous absorption, excluding olfactory stimulation [13]. Blood pressure, breathing rate, and oxygen saturation measurements indicate a decrease of autonomic arousal.

Also neroli EO (that is extracted from flowers of* Citrus *×* aurantium*) demonstrated the capacity to reduce systolic pressure in patients undergoing colonoscopy, even if anxiety was not affected [14].

Finally, it has been demonstrated that also a blend of essential oils, lavender (*Lavandula officinalis*), roman chamomile (*Anthemis nobilis*), and neroli at a ratio of 6 : 2 : 0.5 can reduce anxiety, increase sleep, and stabilize the blood pressure of patients undergoing cardiac stent insertion [15].

## 3. Pain

Perioperative pain is actually well controlled by drugs whose adverse effects are well known. In the control of pain, psychological support techniques can achieve good results by distraction, muscle relaxation, and imagination, thus decreasing the requirements for traditional analgesics and hence reducing the incidence of adverse effects.

In the “distraction hypothesis” any perceived sensory environmental stimulus is sufficient to reduce the pain experienced because the stimulus itself reduces the cognitive resources focusing on pain.

Aromatherapy is one of the potential methods of reducing perioperative pain, but its evidence remains poor.

Preliminary studies have been carried out on the different effects of pleasant and unpleasant smells on pain perception [16]; these studies found unexpected results: both pleasantand unpleasant odors lead to the perception of a greater degree of pain compared to unexposed subjects. This has then been confirmed clinically by Kim et al. [17], who did not find lavender EO effective in reducing pain in patients undergoing breast biopsy. Lavender oil was given as a swab in the oxygen face mask.

Likewise, the efficacy of mandarin EO (*Citrus reticulata*) associated with massage was not proven to be effective to reduce discomfort of babies (3–36 months old) after major maxillofacial surgery [18]. Conversely, a randomized clinical trial on pediatric patients evaluated the effect of lavender EO on pain related to tonsillectomy and found that periodic inhalation of the essence decreased the amount of analgesic required [19].

Good results were obtained also to control pain after laparoscopic gastric banding [20] and cesarean section [21] in a small cohort of patients. The authors concluded that inhaled Lavender essence may be used as a part of the multidisciplinary treatment of pain, but it is not recommended as the sole pain management.

In nonsurgery-related situations, a rigorous study demonstrated that some EOs have analgesic activity (ginger and orange) for a limited period of time; in fact the statistically significant effect observed immediately after application soon wore off. Ginger is one of the most popular herbal remedies and is recommended for rheumatic conditions in Chinese medicine [22].

A recent research added some doubts on the real efficacy of EO in reducing the perception of pain. Masaoka et al. [23], demonstrated that information given to the patients on the lavender effect, the lavender odor itself, and slower breathing contributed to the reduced perception of pain in a controlled study, suggesting a placebo effect. In this apparently well-controlled study, there is one missing information, which is the quality and source of lavender “odor”. It is possible that the lack of efficacy beyond the placebo effect is in fact related to the choice of the EO.

## 4. Postoperative Nausea and Vomiting

Postoperative nausea and vomiting occur as a common side effect of general anesthesia. About one-third of all people undergoing surgery suffer from these conditions at various degrees of intensity. Current therapy has sedation as side effect.

The indication for EOs is mainly limited to ginger (*Zingiber officinale*), spearmint (*M. spicata*), and peppermint (*M.*  ×  *piperita*). Peppermint oil is one of the oldest European herbs used for medicinal purposes. It is a hybrid species of spearmint and water mint (*Mentha aquatica*). The EOs are derived by steam distillation of the fresh aerial parts of the flowering plant. The active ingredients are menthol (35–45%) and menthone (10–30%). Peppermint oil is recommended for its antiemetic and antispasmodic effects on the gastric lining and colon. One possible mechanism of action of peppermint oil in the gastrointestinal system is the inhibition of muscular contractions induced by serotonin and substance P.

Several past studies have shown the efficacy of peppermint in reducing postoperative nausea and vomiting as reported by Tate [24] and Lane et al. [25], in post-cesarean section, where current therapies can interfere with breast feeding. Conversely, a recent Cochrane review [26] concluded that there is currently no reliable evidence for the use of peppermint oil. Similar conclusions were reached by Lua and Zakaria [27] in a review on nausea and vomiting associated with various conditions, not necessarily related to surgery.

After the publication of the review, few other papers were issued. Ferruggiari et al. [28] evaluated the efficacy of inhaled peppermint oil in treating the post-op nausea in a small group of women; their results indicated a good effect of the aroma in reducing the nausea, also compared to standard pharmacological intervention, but statistical significance was not reached due to the small sample of patients.

Recently, Hunt et al. [29] conducted an accurate randomized trial and ascertained that both ginger essential oil and a blend of essential oils of ginger, spearmint, peppermint, and cardamom are effective in reducing nausea and the requirement for antiemetic medications when inhaled following ambulatory surgery.

The use of mint EO has demonstrated applications and advantages as antiperistaltic agent during endoscopy [30] and colonoscopy [31]. In the first study gastric peristalsis was quantified using video-recorded endoscopic imaging and the results confirmed the efficacy of the substance in a dose-dependent manner.

In the second study, premedication with oral administration of capsules of mint EO was beneficial in terms of the time required for cecal intubation and total procedure time, reducing colonic spasm, increasing endoscopist satisfaction, and decreasing pain in patients during colonoscopy.

## 5. Disinfection

The cytotoxic activity of essential oils, mostly due to the presence of phenols, aldehydes, and alcohols, is successfully exploited against prokaryotic cells. Bacteria exposed *in vitro* to different EO show permeabilization of membranes, loss of ions, leakage of macromolecules, and lysis. [32].

The EOs most commonly used for their antibacterial and antifungal properties are the tea tree oil, steam distilled from the leaves, and terminal branchlets of *Melaleuca alternifolia*. In particular the tea tree oil has been shown to be effective *in vitro* on several strains of *Staphylococcus aureus* isolated from wounds (even surgical wounds) and on methicillin-resistant and -sensitive bacteria (MRSA and MSSA) [33].

Its components have shown both bacteriostatic and bactericidal activity *in vitro*. Tea tree oil has also been shown to increase monocytic differentiation *in vitro* and reduce inflammation, therefore assisting the healing of chronic wounds. The main component of tea tree oil, terpinen-4-ol, has been shown to suppress inflammatory mediator production by activated monocytes *in vitro* [33].

Few studies have been published, and some of them, even if encouraging, do not reach sufficient strength, for example, Chin and Cordell [34], who observed a reduction of infected wound healing time when the dressing was treated with tea tree essential oil, or Edmondson et al. [35], who observed a favorable effect of tea tree on wound healing, without negativization of the antibiogram.

In view of this, the general toxicology profile of *M. alternifolia* essential oil suggests that severe reactions would be extremely rare in the absence of ingestion; rare cases of sensitization can be observed probably due to alpha-terpinene, a major constituent in tea tree oil.

The ability of tea tree oil to reduce dermal colonization by methicillin-resistant *Staphylococcus aureus* (MRSA) in critical patients was not proved in a randomized study [36].

Other essential oils have demonstrated *in vitro* activity, but the step from laboratory experimentation to clinical use is not so straightforward.

Promising studies in these files were conducted by Edwards-Jones et al. [39] who demonstrated the *in vitro* potential of essential oils and of their vapors (*Patchouli*, tea tree, geranium, lavender EOs, and commercial mixture grapefruit seed extract) as antibacterial agents for the treatment of MRSA infection. Muthaiyan et al. [40] tested terpeneless cold pressed Valencia orange oil (CPV) for topical therapy against MRSA using an *in vitro* dressing model and skin keratinocyte cell culture model. Warnke et al. [41] tested the *in vitro* efficacy of many EOs (Eucalyptus, tea tree, thyme white, lavender, lemon, lemongrass, cinnamon, grapefruit, clove bud, sandalwood, peppermint, kunzea, and sage oil) with the agar diffusion test, against strains of several common and hospital-acquired bacterial and isolated yeasts and found that thyme white, lemon, lemongrass, and cinnamon oils were effective. The other oils also showed considerable efficacy [42].

Oregano essential oil, which is attributed to antiseptic proprieties by the traditional medicine, has not been tested in clinical trials.

It must be also pointed out that researches currently taking place demonstrate promising *in vitro* results on the synergic activity of EO compared to antibiotics. The *in vitro* inhibitory activity of some antibiotics not only demonstrates additive activity compared to essential oils but also a superior bactericidalcapacity compared to the sum of the activity of the individual substances alone.

Currently, the major applications as antiseptics have been found for infections prophylaxis during small oral surgery, using EOs included in mouthwash, mainly menthol, thyme, and eucalyptol [37, 38].

Data presented are summarized in Table 1, where only “*in vivo*” studies are presented.

## 6. Miscellanea

Essential oils can be successfully applied in few other situations related to surgery.

For example antimicrobial activity of tea tree oil can be exploited also for hand washing. The use of antiseptics is critical in healthcare settings for the prevention of transmission of infections. Messager suggests that tea tree oil-containing hand wash formulations may help reduce the skin carriage of potentially pathogenic organisms also in the surgical environment [42].

Topical application of black pepper may be a viable and effective way to enhance vein visibility and palpability prior to intravenous catheter insertion in patients who have limited vein accessibility. Black pepper essential oil may improve vein access and reduce the need for repeated insertion attempts, thereby reducing patient discomfort and improving patient care [43].

Other promising applications have been proposed for *Helichrysum italicum* as antispastic [44], rose geranium as anti-inflammatory [45], *Origanum majorana* as antimutagenic [46], and many others, but clinical validation is not ready at the moment.

## 7. Final Considerations

Essential oil rigorous studies are still at the beginning and there is some space for new research improving traditional medicine, even from nonoccidental cultures, and transposing it in a modern system where efficacy evidence is the main focus of physicians. Researchers can give their contribution in understanding EO mechanism of actions, as recently proposed by Zhang et al. who demonstrated that the metabolomics approach can capture the subtle metabolicchanges resulting from exposure to EOs [47].

So far, efficacy evidence is contrasting and some literature reviews give very negative opinions like Lee et al. who performed a systematic review stating that ‘‘due to a number of caveats, the evidence is not sufficiently convincing that aromatherapy is an effective therapy for any condition” [48].

It should not be forgotten that essential oils can be contact sensitizers [49] and due to their very complex composition characterized by two or three major components at a relatively high concentration and different substances present in trace, their activity is not completely understood; therefore possible interactions with drugs or peculiar metabolic conditions of surgical patient must be considered [32].

A complete review of the available literature has collected 71 cases of patients who experienced adverse effects of aromatherapy. Adverse effects ranged from mild to severe and included one fatality. The most common adverse effect was dermatitis. Lavender, peppermint, tea tree oil, and ylang-ylang were the most common essential oils responsible for adverse effects, possibly because they are the most commonly used [50]. A case report of seizure related to rosemary EO, possibly secondary to loss of tissue sodium/potassium gradient leading to increased cellular hyperexcitability, must be taken into particular account [51] as well as a case of coma induced by oral long-term abuse and intoxication from methol contained in cough droplets [52].

Finally, it should also be taken into account that there is a trend to use uncommon EOs, often derived from wild plants which have a tendency to produce numerous cultivars with different chemical compositions. Often the different chemotypes have not been tested toxicologically, and possible further problems could derive from this in an uncontrolled market [53].

To conclude, we confirm the need of rigorous clinical trials to disprove the false belief of essential oils as a panacea, and we believe it is necessary that these substances are used at therapeutic level with the same degree of precautions normally followed by the use of pharmacologically active substances.

## Figures and Tables

**Table 1:** Summary of the evidences for the use of EO in surgical patients.

Condition	Essential oil	Number of reference
Anxiety	Lavender	[3, 8–10]
	*Citrus sinensis *	[10–12]
	*Rosa damascene *	[13]
	Neroli	[14]
	*Lavandula officinalis + Anthemis nobilis + Neroli *	[15]

Pain	*Citrus reticulata *	[18]
	Lavender	[19–21]

Nausea	*Menta* × *piperita *	[24–31]
	*Zingiber officinale* + *Mentha spicata* + *Menta* × *piperita *	[29]

Infection	*Melaleuca alternifolia *	[33–36]
	*Menta spicata* +* Thymus vulgaris + Eucalyptus globulus *	[37, 38]
